# Design and performance of a dedicated coherent X-ray scanning diffraction instrument at beamline NanoMAX of MAX IV

**DOI:** 10.1107/S1600577522001333

**Published:** 2022-03-16

**Authors:** Dina Carbone, Sebastian Kalbfleisch, Ulf Johansson, Alexander Björling, Maik Kahnt, Simone Sala, Tomas Stankevic, Angel Rodriguez-Fernandez, Björn Bring, Zdenek Matej, Paul Bell, David Erb, Vincent Hardion, Clemens Weninger, Hussein Al-Sallami, Julio Lidon-Simon, Stefan Carlson, Annika Jerrebo, Brian Norsk Jensen, Anders Bjermo, Karl Åhnberg, Linus Roslund

**Affiliations:** a MAX IV Laboratory, Lund University, 22100 Lund, Sweden; b Microsoft Danmark ApS, Tuborg Boulevard 12, 2900 Hellerup, Denmark; c European XFEL GmbH, Holzkoppel 4, 22869 Schenefeld, Germany; d Axis Communications, Gränden 1, 22369 Lund, Sweden

**Keywords:** hard X-ray nanoprobes, instrumentation, scanning X-ray diffraction, coherent X-rays, X-ray imaging

## Abstract

The diffraction endstation of the NanoMAX beamline is designed to provide high-flux coherent X-ray nano-beams for experiments requiring many degrees of freedom for sample and detector. The endstation is equipped with high-efficiency Kirkpatrick–Baez mirror focusing optics and a two-circle goniometer supporting a positioning and scanning device, designed to carry a compact sample environment. A robot is used as a detector arm. The endstation, in continued development, has been in user operation since summer 2017.

## Abstract 2

The design and the basic performances of the diffraction endstation of the NanoMAX beamline of MAX IV Laboratory in Lund are presented. Designed to perform scanning diffraction, the endstation is adapted to single-particle imaging as well as scanning fluorescence microscopy, tomography, and 2D and 3D ptychography in forward and Bragg geometries, using high-flux nanofocused (coherent) X-ray beams in the energy range between 5 and 28 keV.

## 1. Introduction

NanoMAX, the hard X-ray nanoprobe of MAX IV Laboratory in Lund, Sweden, is designed to exploit the exceptionally low emittance of a 3 GeV storage ring to provide diffraction-limited focused X-ray beams in the energy range between 5 and 28 keV (Johansson *et al.*, 2021 ▸). The set of techniques that the beamline is expected to provide, using both scanning and local approaches, comprises spectro-microscopy, nano-tomography, scanning X-ray diffraction, coherent diffraction imaging, and ptychography in forward and in Bragg geometry. In order to achieve this rather wide scope, two instruments are implemented, tailored to different needs, that provide focused X-ray beams with controlled coherence properties, and complement each other in terms of flexibility, flux and direct resolution. The first in line, the imaging endstation, in commissioning phase at the time of writing, is designed to work in vacuum, to use forward scattering geometry and fluorescence detection, and is aimed at achieving the highest possible direct resolution by the use of diffractive focusing optics, with a small compromise on photon flux. It is designed to achieve stability at the expense of experimental flexibility. The second, the diffraction endstation, placed in a separate experimental hutch, is based on a complementary approach and is at the focus of this article. It provides high-flux focused X-ray beams combined with a flexible setup for (coherent) X-ray diffraction experiments, compromising slightly on direct resolution. Fixed-curvature mirrors are used as focusing devices and a two-circle goniometer allows easy sample change for measurements in air or in a compact sample environment. A robotic detector arm allows flexible detector movement to achieve horizontal and vertical scattering geometries. This is rather unusual for a nanoprobe beamline, where the horizontal scattering geometry, with a reduced number of sample and detector rotations, is preferred for increased stability (Martínez-Criado *et al.*, 2016 ▸; Nazaretski *et al.*, 2017 ▸; Somogyi *et al.*, 2015 ▸; Quinn *et al.*, 2021 ▸). This sets this endstation apart, along with beamline ID01 of the ESRF (Leake *et al.*, 2019 ▸), by which it is inspired, in the landscape of nanoprobe endstations. The instrument provides enough space for hosting compact sample environments, which contributes to its versatility. This endstation has been in user operation since summer 2017 and has produced impactful results in different fields of physics (Rodriguez-Fernandez *et al.*, 2021 ▸; Nukala *et al.*, 2021 ▸; Neckel *et al.*, 2022 ▸), material science (Björling *et al.*, 2020*a* ▸; Dzhigaev *et al.*, 2020 ▸, 2021 ▸; Hammarberg *et al.*, 2020 ▸; Ji *et al.*, 2020 ▸; Marçal *et al.*, 2020 ▸, 2021 ▸; Reimers *et al.*, 2022 ▸; Li *et al.*, 2022*b* ▸) and biology (Silva Barreto *et al.*, 2020 ▸; Gustavsson *et al.*, 2021 ▸), using scanning and local approaches while exploiting scattering, fluorescence and coherent X-ray methods. It has also received considerable interest from communities of soft-matter physics (Nissilä *et al.*, 2021 ▸; Huss-Hansen *et al.*, 2022 ▸) and geology (Warlo *et al.*, 2022 ▸) and has recently produced the first results of 3D ptychography in Bragg, demonstrating the great opportunities provided to crystal microscopy by the high coherent flux of fourth-generation sources (Li *et al.*, 2022*a* ▸). In the following, we illustrate the concepts at the basis of the diffraction endstation design, the engineering solutions used to guarantee stability while preserving flexibility, and its performance. For details about the NanoMAX beamline layout and optics we refer the reader to Johansson *et al.* (2021 ▸).

## 2. Infrastructure

**Figure 1:**
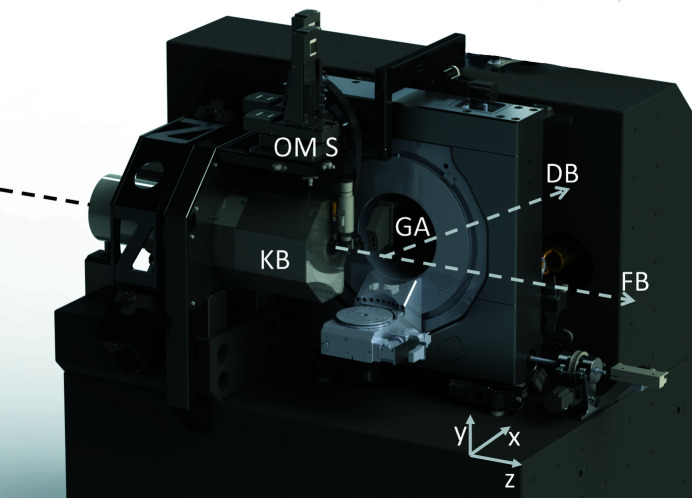
A 3D rendering of the diffraction endstation with laboratory coordinate system (*xyz*). The granite block at the heart of the instrument provides support to the mirror mechanics (KB), the goniometer assembly (GA) and the optical microscopes support (OMS). The directions of the forward X-ray beam (FB) and the diffracted beam (DB) are marked by dashed lines.

Stability is certainly the main concern in the design of a nanoprobe instrument. The best way to ensure stability for such an instrument is to strongly couple the source and sample and separate this unit from external disturbances. For a synchrotron nanoprobe, however, this approach is made impossible by the exceptional distance between primary source and sample, which, for the diffraction endstation, is 98 m. In this case, the requirement of strong coupling is transferred to the sample–focusing optics unit instead. The core of the instrument is a large granite block hosting the focusing optics and the sample support, as shown in Fig. 1 ▸. Detectors and other auxiliary equipment, as described in this article, are mechanically decoupled from this unit to preserve its stability.

Reducing sources of instability relies strongly on controlling them. The two main sources of instability are mechanical vibrations, responsible for high-frequency (sub-second) motion, and thermal or mechanical drifts, responsible for slow or long-term (from many seconds to hours) movements. To reduce the effect of vibrations, the beamline from source to endstations was built on the same stabilized floor as the 3 GeV ring (Tavares *et al.*, 2014 ▸; Johansson *et al.*, 2021 ▸). Measurements of floor stability indicate an amplitude of the vertical and horizontal vibrations well below 10 nm RMS, integrated for all frequencies above 5 Hz and a characteristic frequency of ∼14 Hz (not shown here). To keep control of the vibration level of the floor and all components fixed on it, a very high stiffness is required for all installed equipment, with mechanical eigenfrequencies above 50 Hz, three times larger than the natural resonance frequency of the floor. This condition is applied to all purchased and built equipment, where possible. All mechanical supports and platforms (granite blocks and metallic frames) are meant to *lift* the floor stability to the equipment and the X-ray beam level. Therefore, similar requirements about stiffness and eigenfrequencies are applied to all supports.

**Figure 2:**
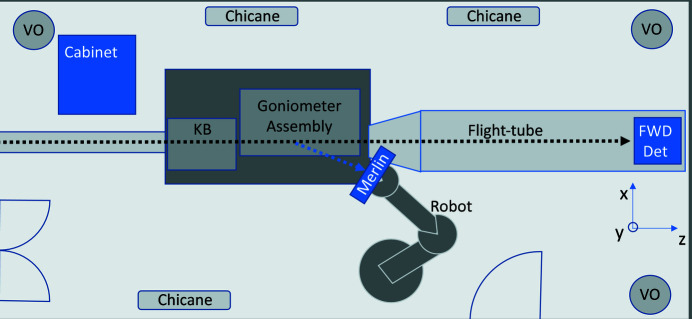
Layout of the experimental hutch hosting the diffraction endstation at NanoMAX. The directions of the forward and diffracted X-ray beams are marked by dashed lines. The ventilation outlets (VO), the inside electronic cabinet, the chicanes used for the cables, the focusing mirrors (KB), the goniometer assembly, the robot detector holding the Merlin detector, the 2D forward detector (FWD Det) and the flight tube are marked.

To prevent long-term drifts, the temperature stability of the large experimental hutch, where the endstation is placed, is controlled to 0.1°C by an air conditioning system based on laminar flow. When possible, the power supply units for electrical equipment, as well as controllers and water cooling units for detectors, are kept outside the experimental hutch and connected to the instruments by long cables passing through designated chicanes. For a number of devices, however, for which a limited cable length is required (*e.g.* piezo actuators, detector electronics), the controllers are hosted in a cabinet placed inside the hutch (*cf*. Fig. 2 ▸) that is thermally controlled. The actual temperature variations measured on different parts of the instrument during operation show a thermal stability in the 0.02–0.05°C range (Johansson *et al.*, 2021 ▸).

## 3. Focused X-ray beam

The X-ray beam at the diffraction endstation is focused by a fixed-curvature Kirkpatrick–Baez (KB) mirror system (JTEC, Japan). The KB mirror specifications comply with the request of providing a distance of at least 100 mm between the last physical component of the beamline and the sample position, at the focal plane, to guarantee enough space for all the sample degrees of freedom necessary for diffraction as well as for auxiliary equipment, with a small compromise on the achievable size of the beam. The result is a fixed numerical aperture (NA) optics producing a diffraction-limited focus at the sample position, at 98 m from the source, with size varying from 200–40 nm in the energy range 5–28 keV. This is achieved using the NanoMAX secondary source aperture, positioned at 51 m from the undulator, as a virtual source. The secondary source aperture is also used to control the coherence properties of the X-ray beam. Further details on the optical properties of the KB mirrors can be found in the work of Johansson *et al.* (2021 ▸).

### 3.1. KB mirror mechanics

**Figure 3:**
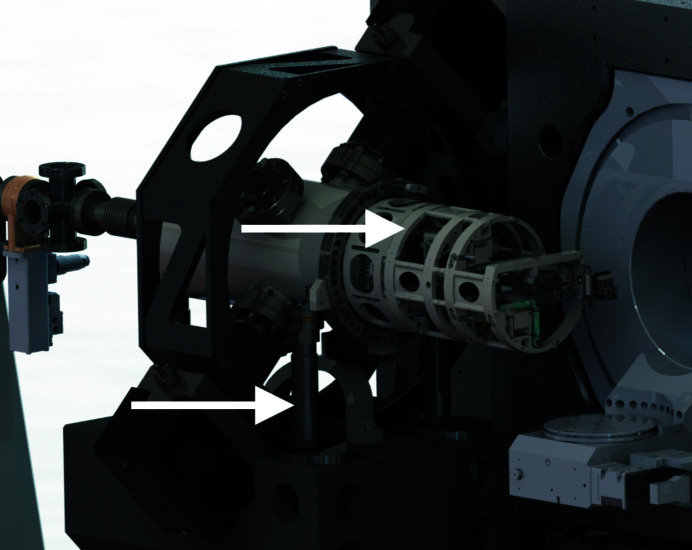
A 3D rendering of the KB mirror system, the body of the chamber partly hidden for visibility of the internal parts. The two arrows indicate, from left to right, one supporting leg and the light cage hosting the mirrors. In operation conditions, the whole system is under vacuum.

The main criterion for the design of the KB mirror support is the reduction of the number of stages, by the use of parallel mechanics. Avoiding a stack of translation and tilting stages helps reduce parasitic motion and improves overall stability. Following this idea, the two mirrors are treated as a single unit placed on a long-range positioning system, while a fine tweaking applies to each mirror separately. The mirrors are mounted in a light and stiff supporting cage, shown in Fig. 3 ▸, where six manual screws and three piezoelectric actuators are used for the fine adjustment of their tilt angles and their mutual position.

This unit is placed in a vacuum chamber supported by an alignment system according to the MAX IV standard with three vertical, two transversal and one longitudinal support. The vertical and transversal support legs consist of sturdy fine-threaded rods, nuts and spherical washers that provide exceptional stiffness to the system. One leg unit is indicated by an arrow in Fig. 3 ▸. The longitudinal support is fixed without adjustment.

The capability of pre-positioning the mirrors inside their support with a precision of 50 µm for the mutual distance and 100 µrad for the mutual rotation angles (mirror pitch, roll and yaw) provided by the MAX IV alignment team is a crucial parameter of the KB mechanics design. The alignment system provides up to 10 mm range in vertical and horizontal translations and up to 20 mrad tilts, and precision better than 5 µm and 10 µrad, respectively, sufficient for coarse positioning and alignment. The piezo actuators used for the fine adjustment of the mirror tilt angles have a total stroke of 30 µm and a nanometre precision. They provide control of the mirror pitch angles over a range of ∼350 µrad with a precision of a few nanoradians, necessary for the fine tweaking of the mirror pitch angles to reach a perfect alignment of the vertical and horizontal foci. For each mirror, the pivot point for tilt and pitch angles is the edge opposite to the piezo actuator. Due to the limited stroke of the actuators, combined with the limited length of the mirrors (Johansson *et al.*, 2021 ▸), this design choice greatly increases mechanical stability when compared with a system with a central pivot point, with virtually no impact on the X-ray beam position on the mirrors.

**Figure 4:**
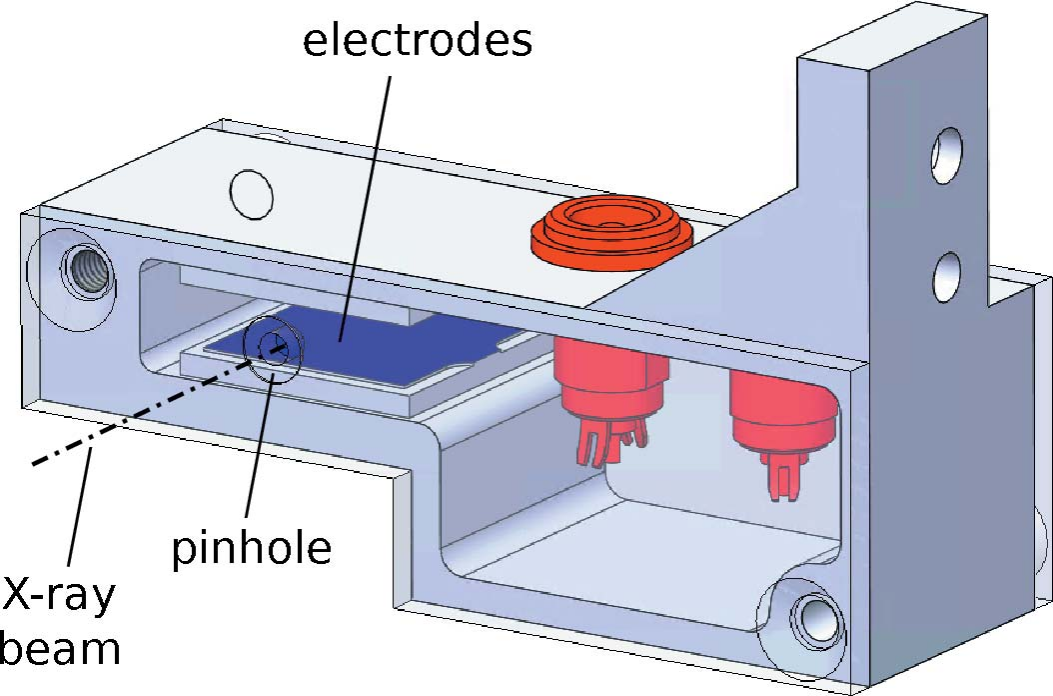
A rendering of the ion chamber. Parallel plate electrodes (blue), a recess for mounting a pinhole (identical recesses are found on both the incoming and outgoing sides) and the outgoing X-ray beam are marked. The electrical connectors are in red and the internal cabling is omitted.

The illumination of the KB mirrors is defined by a set of four motorized slit blades, which are installed 50 mm upstream of the mirror pair. The beam-defining edge of each slit blade is made of a 1 mm-thick W rod (Le Bolloc’h *et al.*, 2002 ▸), which is inserted into a milled-out channel in a 2 mm-thick W block and is fixed with a small amount of In to avoid any outgassing from a polymer-based adhesive. The exit window of the mirror vacuum chamber is made of a both-side-polished single-crystal chemical-vapor-deposited diamond with a diameter of 4 mm and a thickness of 50 µm, which is brazed into a DN CF16 vacuum flange (Applied Diamond Inc., USA). A 200 µm pinhole (Pt/Ir) is placed on a compact *xyz*-translation stage (SmarAct GmbH, Germany) at a distance of ∼1/2 of the focal length of the mirrors to clean spurious scattering from the mirror edges and cut the purely reflected beam. A compact custom-built ion chamber with a path length of 15 mm is integrated in this unit, allowing a measurement of the incoming photon flux, which is useful for normalization of the signal. The two parallel Cu electrodes, with a thickness of 0.2 mm and surface of 15 mm × 15 mm, placed at a distance of 2 mm, have an applied voltage of 180 V. A drawing of the ion chamber is shown in Fig. 4 ▸.

### 3.2. Mirror alignment and stability

Ptychography measurements of a test structure are routinely used to optimize the KB mirror alignment prior to every experiment as well as to check it at regular intervals during the experiment. The X-ray wavefront retrieved at the sample position is propagated for several hundreds of micrometres in opposite directions along the beam axis to find the focal plane of the horizontal and vertical mirrors. Small adjustments of the pitch piezo motors are then applied to each mirror to make the focal planes of the two mirrors coincide at the sample position. To provide a numerical value for this tweaking, a shift of the focal plane along the beam axis of 100 µm is corrected by a contraction of the mirror pitch piezo actuator by 72 nm for the vertical focusing mirrors, or by 117 nm for the horizontal one. Results from the ptychography inversion are also used to characterize the coherence of the beam. At an energy of 8 keV, the flux available in a beam that can be considered fully coherent at the diffraction endstation, is ∼6 × 10^10^ photons s^−1^ in a beam with a width of 110 nm (full width at half-maximum), with an electron-beam current of ∼300 mA in the storage ring. If partial coherence is acceptable, the flux exceeds 10^11^ photons s^−1^ (Björling *et al.*, 2020*b* ▸; Johansson *et al.*, 2021 ▸).

**Figure 5:**
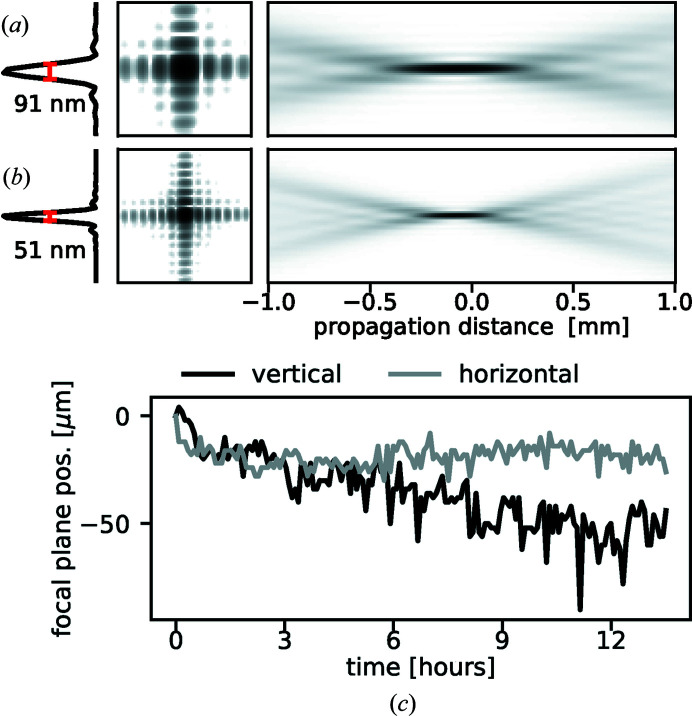
Beam profiles at (*a*) 10 keV and (*b*) 18 keV. The profiles in focus are determined by ptychographic imaging 300 µm out of focus and subsequent numerical wavefront propagation. The in-focus images (left) show intensity on a log scale, while the propagation images (right) show side views of the beam intensity on a linear scale. (*c*) The movement of the focal planes of the two mirrors along the beam propagation, measured with respect to their initial positions, during a 13.5 h experiment at 10 keV. At this energy, the depth of focus of the X-ray beam is 330 µm.

**Figure 6:**
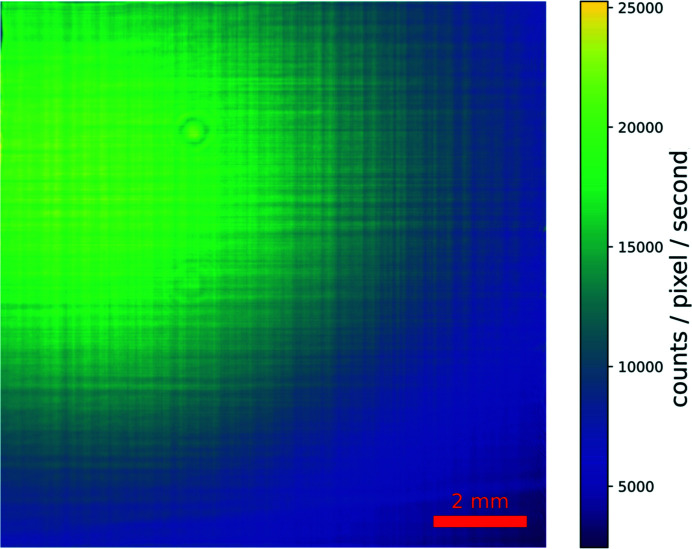
A far-field image of the transmitted beam as measured on a high-resolution sCMOS camera (ANDOR Zyla 4.2, 6.5 µm pixel size) coupled with a 10× magnification optical element and a 10 µm-thick LuAG:Ce fluorescence screen. The camera was positioned at 1.12 m from the focal plane and the beam fills the whole camera field of view. The scale bar refers to the camera dimensions. The uniform distribution of intensity is an indication of the great quality of the KB mirror surfaces.

Figures 5 ▸(*a*) and 5 ▸(*b*) show the beam profile in the focal plane and propagated along the beam axis, retrieved with ptychography at two energies, obtained using a coherent illumination of the KB optics (Johansson *et al.*, 2021 ▸; Björling *et al.*, 2020*b* ▸). The measured beam size corresponds to the predicted value for a diffraction-limited focus at the corresponding photon energy and mirrors NA that, by design, is set to be similar in the horizontal and vertical directions (Johansson *et al.*, 2021 ▸). This is NA = *h*/2*f* = 6.2 × 10^−4^, where *h* is the acceptance height of either mirror and *f* is its focal length, corresponding to a beam divergence of ∼1.2 mrad. The absence of distortions in the high-resolution far-field image of the transmitted beam in Fig. 6 ▸ gives further confirmation of the quality of the focused beam and of the mirror surface.

Figure 5 ▸(*c*) shows the position of the X-ray focal plane over more than 13 h of repeated ptychographic imaging, directly after a period of having the hutch door open to rebuild the setup. For each of the 160 scans, the probe was retrieved and propagated numerically. The focal planes of the horizontally and vertically focusing mirrors were estimated individually by finding the planes of maximum sum-squared intensity in each direction. A slight thermal settling of the foci can be seen to occur, amounting to a total of ∼50 µm in the vertical direction and 15 µm horizontally. During the second half of the run, the RMS fluctuations of the focal positions along the beam were 10 µm (vertical focus) and 4 µm (horizontal focus), much smaller than the focal depth of ∼330 µm at 10 keV. A shift of the vertical (horizontal) focal plane of 10 µm along the beam propagation corresponds to a pitch-angle variation of ∼87 nrad (∼138 nrad), and a resulting lateral movement of the beam in the focal plane of ∼50 nm. At 10 keV this is approximately equivalent to 50% of the beam size, again highlighting the compromise between a flexible optical design with a long working distance and direct resolution. This value is also comparable with the sample stability provided by the goniometer stage during a typical rocking curve rotation (*cf.* Section 4.1 below). The stability of the beam position in the focal plane with varying energy has been characterized elsewhere (Osterhoff *et al.*, 2019 ▸). The beam was found to be stable within 100 nm when varying the photon energy.

## 4. Sample

The combination of a flexible diffraction geometry with a nano-focusing setup presents enormous challenges for the preservation of the stability and the reproducibility of the sample movements. The compromise offered by the diffraction endstation design, following a concept developed elsewhere (Leake *et al.*, 2019 ▸), is the reduction of the sample rotations to two stages and the decoupling of the detector arm, here replaced by a robot. A high-precision and high-load piezo-based assembly is used to position and finely scan the sample in the nano-focused beam. The assembly provides the sample with all translation and rotation degrees of freedom needed for single-crystal scanning diffraction, which is a rare occurrence in the world of nanoprobe instruments (Leake *et al.*, 2019 ▸).

The sample stage is designed to capitalize on one of the greatest advantages of hard X-rays, *i.e.* their compatibility with sample environment due to their large penetration depth through light-material windows. A space is therefore made available for a light (≤1 kg) and compact (≤80 mm) sample environment on the top of the two goniometer rotations and the three-axis scanning stage. Because the requirements on sample environment are linked tightly to the very specific science case of each experiment, no fixed set of sample environments is offered to the users. Instead, fitted solutions for every case are designed by the users with help and input from the beamline team. Using this approach, various sample environments have been developed and used at the diffraction endstation, some of them producing already published results. These include an electrochemistry cell (Björling *et al.*, 2019 ▸), a diamond anvil cell (Ji *et al.*, 2020 ▸), a heating and a Peltier cooling stage, and an environment for controlled atmosphere. More complex sample environments have also been developed by user groups at their home laboratories, to be compatible with the diffraction endstation setup. These include an *in situ* atomic force microscope (Lund University), a nano-indenter (Chalmers University) and an *in situ* chemical reactor based on commercial micro-electro-mechanical systems (MEMS) chips.

### 4.1. Goniometer

**Figure 7:**
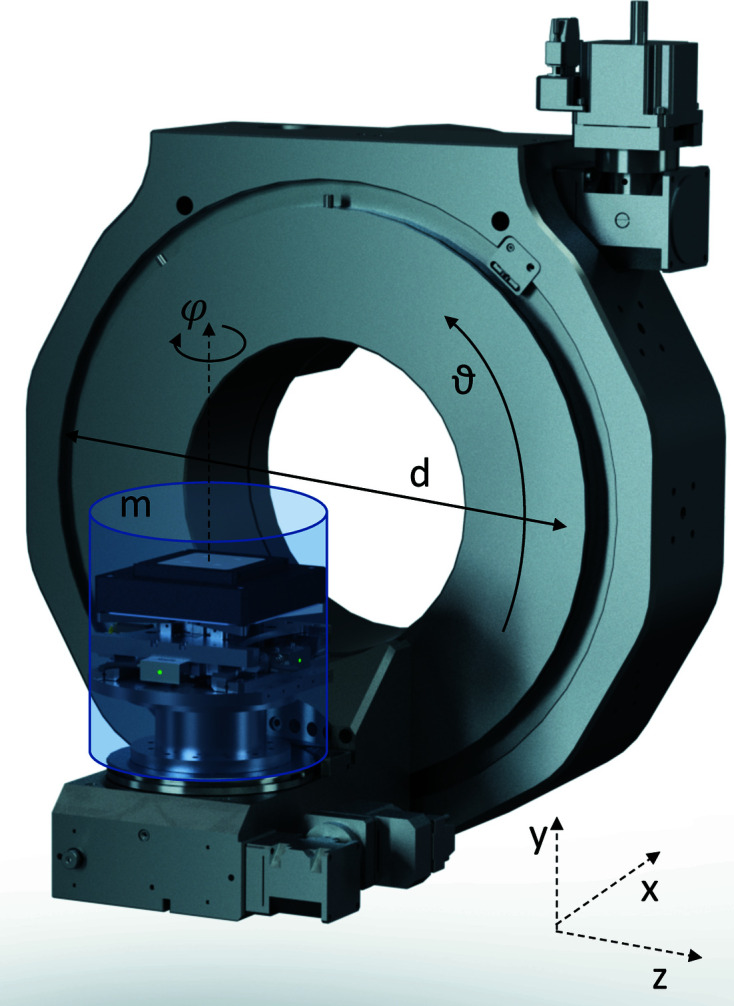
A 3D rendering of the two-circle goniometer assembly. The ϑ and φ rotations are highlighted. The transparent cylinder represents the volume envelope available for the sample scanner and sample environment. For a torque-free assembly, the total mass *m* should be 5 kg.

**Figure 8:**
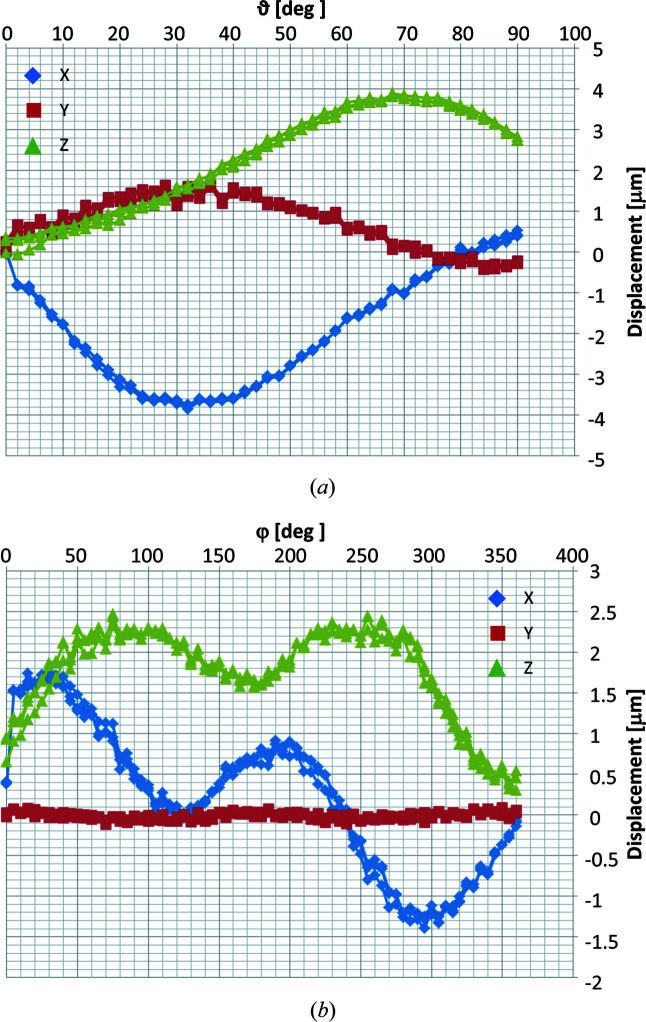
Measurement of the sphere of confusion of the goniometer circles (*a*) ϑ and (*b*) φ, evaluated from the lateral displacement of a polished sphere placed on a rigid support in the center of the assembly. Three repetitions of a full rotation are performed for each circle, separately. Repeatability of the movement is visible from the overlap of the data points. (Courtesy of Huber GmbH & Co., Germany.)

The two-circle goniometer of the diffraction endstation is a ϑ–φ assembly produced by the company Huber (Huber GmbH & Co., Germany) and is shown in Fig. 7 ▸. It should ideally provide a 360° rotation of the azimutal angle φ and a rotation of ϑ in the range [−2°, 90°] with a few tens of nanometres runout, as well as supporting a positioning and scanning stage and a compact sample environment. These requests exclude the use of air-bearing rotations, which, while capable of providing sub-50 nm rotation axis runout, cannot be mounted in a diffraction-compatible assembly and often underperform during angular motions in small steps. The compromise proposed here is the requirement for the goniometer circles to have an Abbe error (or runout) of 60 nm, ∼50% of the beam size at the energy providing optimal flux, within a rotation of only 2°, *i.e.* the typical range for a rocking scan around a Bragg peak, at any angular position, while showing reproducible lateral displacements in the full rotation range (*cf.* Fig. 8 ▸). This is achieved using high-precision standard rotation stages with intersecting axes and a sphere of confusion of only 6 µm in diameter for each circle and of 10 µm for the assembly, limited by the precision of the mechanical mounting. The goniometer assembly is based on a modified design of standard components to improve stability and reduce torque: the overall shape of the supports and counter weights and its mass distribution is such that the center of mass of the assembly coincides with the geometrical center of the large circle ϑ in Fig. 7 ▸. Characterization of repeatability and sphere of confusion of the circles ϑ and φ, measured independently via the lateral displacement of a sphere positioned in the center of rotation (CoR) during three repeated rotations of each circle in their working range, is illustrated in Fig. 8 ▸. Repeatability of the movement is uni-directional for both rotations, *i.e.* it is achieved while approaching a specific angular position from the same direction. In the operation, this is achieved by introducing a relatively large (0.25°) backlash in the motion-control software that is applied when approaching a certain angular position from larger values.

The sphere of confusion is well within specifications: the circle φ shows a runout of ∼30 nm per degree over the whole investigated range while the circle ϑ shows a runout of ∼60 nm per degree, see Fig. 8 ▸. These are comparable with the position stability of the beam in the focal plane. This implies that scanning diffraction measurements, requiring full 3D reciprocal mapping, that need a lateral resolution better than 60 nm must rely on realignment procedures, or can be performed, instead, exploiting X-ray energy scans (Cornelius *et al.*, 2011 ▸). When using digital microscopies, based on the inversion of 3D coherent data in Bragg geometry, to reach the best achievable resolution, single-angle approaches (Hruszkewycz *et al.*, 2017 ▸) or advanced inversion routines (Li *et al.*, 2022*a* ▸) should be considered. The ϑ stage is intrinsically a full circle and the limits in its angular motion are imposed by the diffraction setup and possible collisions, and can be slightly surpassed, if necessary.

The goniometer support is designed and produced in-house. In analogy with the design of the KB mechanics, it is based on parallel mechanics to reduce instabilities and keep a minimum lateral encumbrance. This last aspect is especially important, as this support must keep the CoR of the large goniometer assembly, a total mass of 250 kg and lateral size of 450 mm, within 100 mm from the KB mirror chamber, following the basic design requirement. With the implemented solution, the support frame is positioned by five micrometre-precision screws. These screws are the same used for the fine adjustment mechanics of the girders holding the magnets of the 3 GeV ring multi-bend achromat (MBA) units (Tavares *et al.*, 2014 ▸). They are motorized and use a built-in linear encoder. Crucially for this instrument, the parallel design also allows a very fine adjustment of the orientation of the ϑ and φ rotation axes with respect to the direction of the focused X-ray beam, which exits the KB mirrors assembly in a direction *z*′ tilted by ∼5.4 mrad in the horizontal direction and 5 mrad in the vertical direction with respect to the incoming beam. One last screw allows the adjustment of the goniometer position along this direction *z*′ and for a translation up to 200 mm downstream of the focal plane. This allows the positioning of the goniometer CoR far beyond the X-ray beam focal plane, to position the sample out of focus without moving the optics, while leaving the sample in the CoR of the goniometer. This can be useful for measurements requiring a curved wavefront and/or a wider beam, and creates space for the introduction of new focusing optics without the need for removing the KB support.

In order to reduce torque on the goniometer support, the screws have a sliding interface with it. Gravity in the vertical direction and two large springs pulling against the granite block in the horizontal direction guarantee the stability of the support in static operation. Besides providing great stability in a reduced space, the advantages of using this solution are the simplicity of its design, the low cost and its flexibility. This comes at the cost of a complex procedure for the initial instrument alignment, which requires the expertise of the alignment team. This operation, however, is repeated in a seldom way, with the purpose of checking the stability and the reliability of the alignment, thus it does not represent an issue for daily operation. Once the alignment of the CoR with the beam position is completed, successive realignment is performed by small tweaking of the KB mirror pitch, which provides a precision well below the micrometre level accessible with the positioning screws.

### 4.2. Sample positioning and scanning

**Figure 9:**
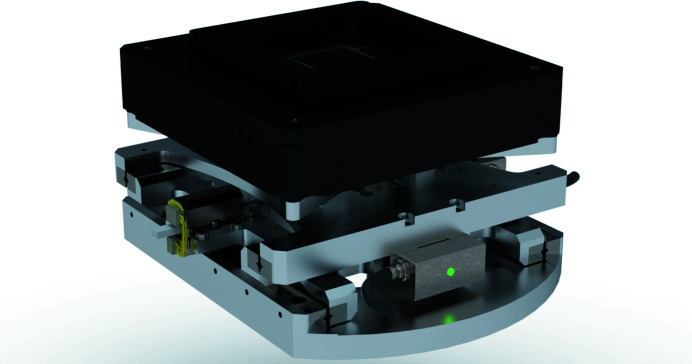
The sample coarse-positioning assembly, in gray in the figure, carries a high-precision three-axis scanner from nPoint, in black.

The sample-positioning and scanning unit of the diffraction endstation has been developed in-house and is shown in Fig. 9 ▸. Three perpendicular long-range piezo crawling translations (LEGS Linear LT40, PiezoMotor) are nested in a lightweight compact support. This requires less space than an assembly of commercially available positioners of similar mechanical specifications. The in-house design provides a stiff assembly capable of 16 mm range for the positioning of the sample and 2 kg of load. The built-in linear encoders have a resolution better than 10 nm; however, the repeatability is limited by the step size of the piezo crawlers to ∼100 nm (*cf.* Table 1 ▸). A better repeatability can be achieved, when needed, by using smaller step sizes at the expense of motion speed. The performance of the sample support does not appear to be dependent on the actual position of the goniometer. However, it is experimentally confirmed that the rigidity of the assembly is such that the sample stability is well within the beam size, for movements of the tilt angles up to 30°. A three-axis high-precision piezo scanner (NPXY100Z100-135, nPoint), with a stroke of 100 µm for each axis and a 1 nm precision, is used for sample fine motion and scanning. This scanner allows continuous acquisition schemes that, by being extremely time efficient, can help counteract effects of sample drift during the measurements. At the time of writing, the support has been in place for a limited time and a complete characterization of its performances is ongoing. Improvements in the motion reproducibility and the mechanical stability are clearly noticeable with respect to an earlier temporary solution that included a compact scanner from PI (P-611.3 NanoCube) mounted on a stack of motorized linear stages. A breadboard-like support (M3 holes in a square grid with a 5 mm period) is fixed on the nPoint scanner to provide flexibility for the sample mounting. A variety of sample supports are available at the endstation, including pins, flat-end stubs and comb-like supports, like the one shown in Fig. 11.

### 4.3. Piezo rotation stage for nano-tomography

**Figure 10:**
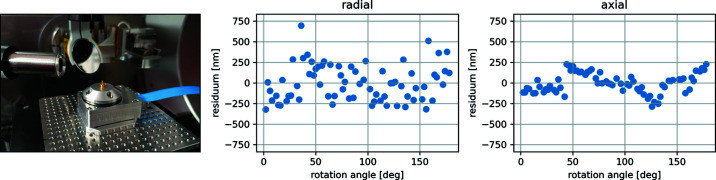
(Left) A piezo rotation stage mounted on top of the piezo scanner breadboard with a pin holding the sample for a nano-tomography experiment. (Center) Estimated position error in the radial and (right) axial directions. Both measurements show the resulting sum of all contributions: that is, the position errors from every stage in the stacks, as well as long-term drifts in the whole setup.

Due to the rather large sphere of confusion (*6* µm for a full rotation), the φ circle of the goniometer assembly is not adapted to perform nano-tomography measurements. Therefore, in anticipation of the dedicated endstation, a compact piezo rotation stage has been implemented in the diffraction endstation allowing pioneering nano-tomography studies to be successfully performed at NanoMAX (Kahnt *et al.*, 2020 ▸). The rotation stage (Xeryon, XRT-A-25-109) can be mounted on the top of the stack of sample positioning stages. This has the advantage that the required lateral scanning movements can be performed without the need for coupling the motion of multiple physical axes. Having the rotation stage last in nano-tomography experiments has the disadvantage of having to correct for the eventual off-center mounting of the sample using the translation stages below the rotation stage. We have successfully tested the use of both the coarse base stages (Kahnt *et al.*, 2020 ▸) and the piezo scanner stages to account for large (>10 µm) and small off-center sample mountings, respectively. Using the extracted relative shifts between acquired tomographic projections and subtracting a fitted sinusoidal, we verified that the axial and radial error motions are in the order of 0.5 µm (see Fig. 10 ▸), which matches the stage’s specifications and is sufficiently small for nano-tomographic experiments of micrometre-size samples.

### 4.4. Optical microscopes

**Figure 11:**
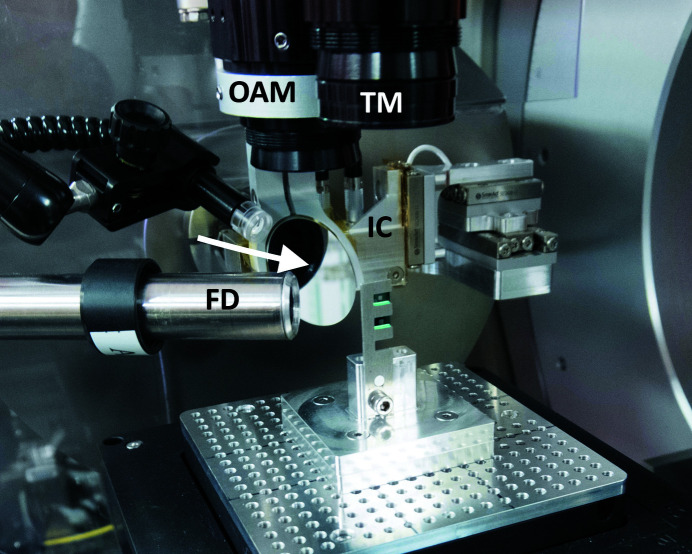
A close-up photograph of the sample stage: the top microscope (TM), the on-axis microscope (OAM), the 45° mirror with the central hole indicated by the white arrow, the ion chamber (IC) and the fluorescence detector (FD) are marked. The comb-like sample support is designed for X-ray fluorescence and/or transmission measurements. The breadboard fixed on the sample scanner provides flexibility for sample mounting.

Sample microscopes improve the user experience and allow a rapid recognition of the region of interest on the sample, which is necessary when signals from X-rays are not reliable enough for sample pre-alignment. The diffraction endstation is equipped with two optical microscopes for micrometre-precision sample positioning and alignment, as shown in Fig. 11 ▸, both fitted with a Navitar 12× Zoom Lens System. The on-axis microscope is coupled with a 45° mirror with a 0.6 mm center hole for the transmission of the incoming X-ray beam. This allows for the inspection of the sample from the perspective of the X-ray beam while measuring. It has a working distance of 86 mm, a maximum resolution of 3.3 µm and a depth of focus smaller than the KB mirrors provide. Therefore, the optical focal plane can be used as a reference for the X-ray focal plane. The field of view can be adjusted between 5.848 mm × 4.678 mm and 0.484 mm × 0.387 mm by a motorized zoom^1^. The top microscope looks at the sample along the *y* direction. It supports the easy positioning of a sample in the CoR of the goniometer. The top microscope has a working distance of 50 mm and a maximum resolution of 2.2 µm. Its field of view can be adjusted between 3.898 mm × 3.119 mm and 0.323 mm × 0.258 mm by a motorized zoom.

Each optical microscope is mounted on an individual stack of three linear stages (OWIS LIMES 84N-45-IMS) for precise *xyz* positioning and alignment with respect to the X-ray focal point. The optical microscopes are essential tools in the initial alignment, and the successive inspection, of the goniometer CoR with respect to the X-ray beam. They also play a central role in the routine adjustment of the KB mirrors carried out with ptychography, providing a rather precise placement of the sample in the ideal position for the X-ray focal plane; namely, the CoR of the goniometer. Further alignment of the sample in the beam is easily performed using the relevant X-ray signal.

## 5. Detectors

The diffraction endstation has three photon-counting pixel detectors with Si sensors: a Merlin Si Quad from Quantum Detectors, with 516 × 516 pixels of 55 µm edge size and 500 µm sensor thickness, used mainly for diffraction experiments; an Eiger2 X 4M from Dectris, with 2968 × 2162 pixels of 75 µm edge size and 450 µm sensor thickness; a Pilatus2 1M, also from Dectris, with 1475 × 1679 pixels of 172 µm edge size and 500 µm sensor thickness, both used for scattering experiments in forward geometry.

**Figure 12:**
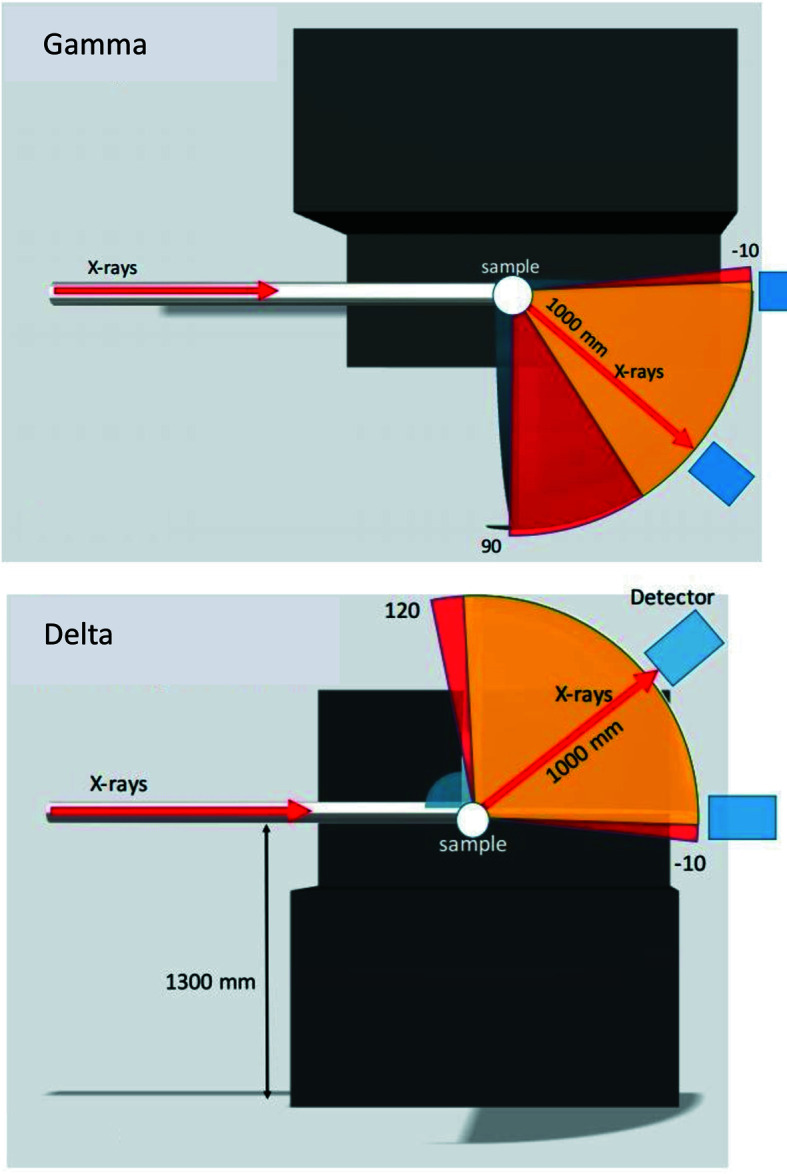
Schematics of the angular range available for the robot arm in the horizontal (gamma, from a top view) and vertical (delta, from a side view) directions. The yellow and red areas represent the minimum and the optimal requirements for the accessible angular range, respectively. The minimum sample–detector distance is 250 mm, while the maximum is 1000 mm in the whole range and can go up to 1200 mm in a small angular range close to the forward beam direction.

**Figure 13:**
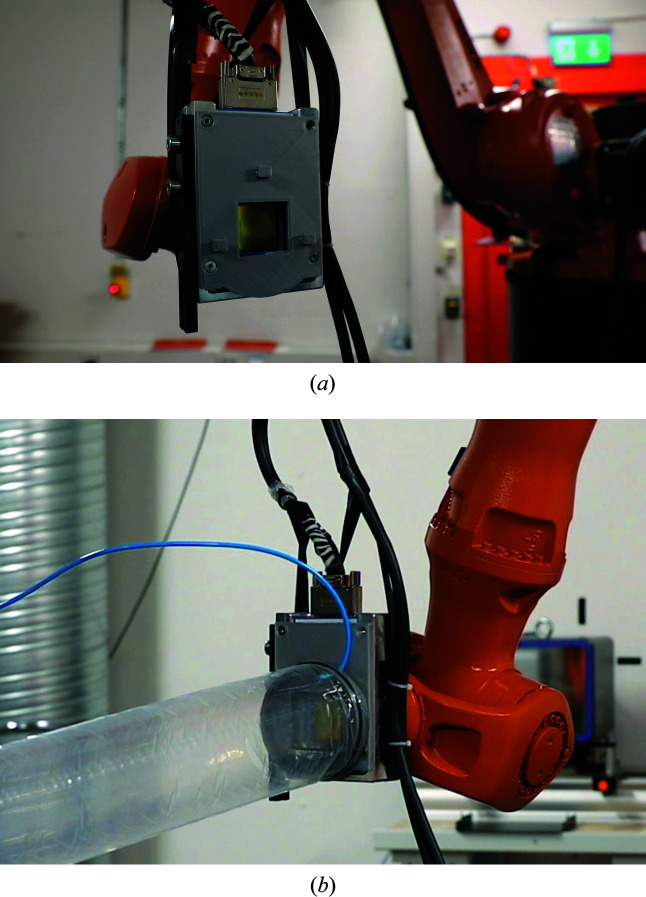
Photographs of the Merlin pixel detector mounted on the robot arm. (*a*) Using the three hooks visible around the central chip, (*b*) the light He-flushed flight tube is mounted on a plastic frame directly on the detector. It is positioned between the sample and the detector to reduce air scattering.

The light and compact Merlin detector is mounted on a robot arm (Cybertech KR20 R1810, Kuka), set up to position the detector in a spherical coordinate system centered on the goniometer CoR, with polar (δ) and azimuthal (γ) angles in the range [−5°, 90°], and radius (distance from the sample) variable in the range [250 mm, 1000 mm] (see Fig. 12 ▸). The two angles and the sample-to-detector distance are calibrated using the diffraction signal from an Si powder sample measured at different energies. The robot’s native accuracy is improved with the use of a calibration software, developed in collaboration with the Department of Automatic Control of Lund University and Cognibotics, a spin-off company from Lund University, and reaches 20 µm for absolute positioning precision and less than 10 µm for repeatability, well adapted to the pixel size of the Merlin (Bring, 2018 ▸). The pixel size and the sample–detector distance achievable provide an angular resolution in the range [0.003–0.015°]. Conversely, the angular range that the detector can cover in a fixed position spans in the range [1.5–7.7°]. This provides flexibility in terms of resolution versus range achievable in reciprocal space. The Merlin detector is normally used in continuous mode, where it can achieve 300 Hz frame rate. Alternatively, it can also be used in burst mode, where it is capable to achieve up to 1 kHz frame rate for one-second periods, limited by the local buffer capacity, and is subject to future upgrades. This functionality has already proven to be useful in a few experiments (Nukala *et al.*, 2021 ▸). Photographs of the detector arm assembly are shown in Fig. 13 ▸. A light plastic inflatable flight tube can be installed on the robot, via a 3D-printed interface, between the detector and the sample, and filled with He gas to reduce air scattering and improve the signal-to-background ratio for diffraction experiments.

**Figure 14:**
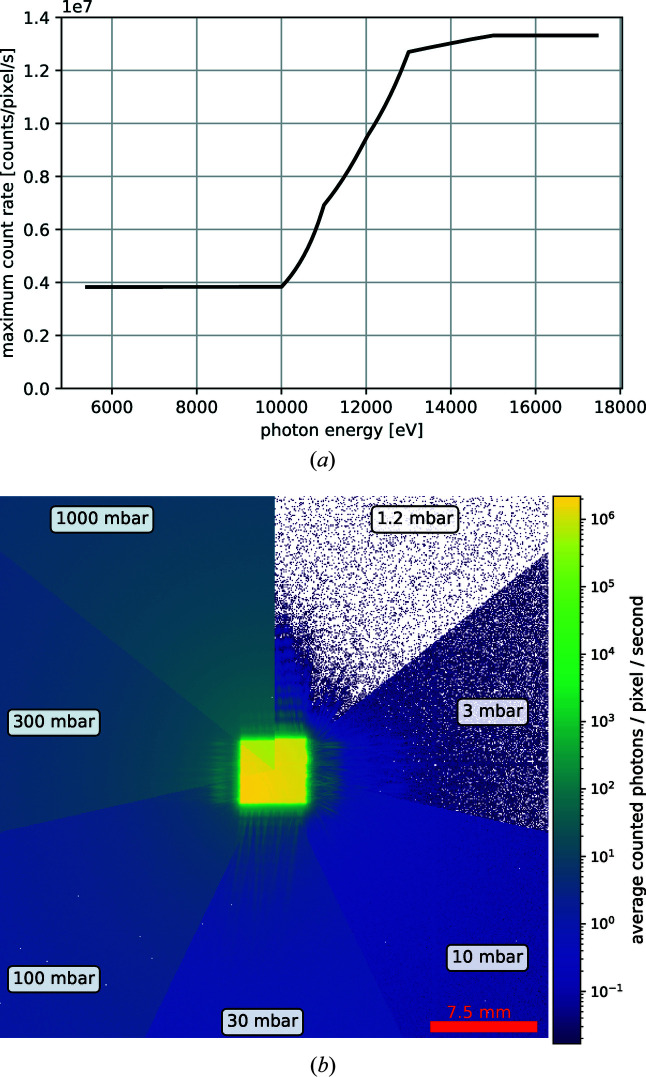
(*a*) Maximum count rate per pixel of the Eiger2 X 4M depending on the incident-beam photon energy. (*b*) Improvement of the (air-)scattering background with the reduction of pressure inside the flight tube, housing the Eiger2 X 4M detector. The presented data are frames averaged over 180 s. The images show the transmitted beam on the detector at 3.51 m distance from the focus, with a photon energy of 12.4 keV and a secondary source aperture of 8 µm (horizontal) × 5 µm (vertical).

A large area detector (Dectris Eiger2 X 4M or Dectris Pilatus2 1M) is used to collect data in forward scattering geometry. It can be mounted close to the sample and be used together with a beam stop for recording diffraction patterns in wide-angle scattering geometry. The minimum distance to the beam focus is 140 mm. For such experiments, an He-flushed plastic flight tube, mounted with a custom 3D-printed holder on the detector, can be used to reduce the air-scattering background (see Fig. 13 ▸). For a position further downstream, the detector is flexibly mounted on a detector table on two supports that allow independent motorized vertical and horizontal translations, and thus also an alignment in pitch and yaw. Longitudinally, the detector table can be manually adjusted and fixed in place. The Eiger2 X 4M detector can be used either in air or, preferably, inside a modular vacuum flight tube. In the latter case, the detector can be used without its window, making the entry window of the flight tube (5 mm × 5 mm × 0.001 mm of Si_3_N_4_) the only window between the sample and the detector panel. The vacuum in the flight tube strongly reduces the air-scattering background [see Fig. 14 ▸(*b*)], which allows one to record signal from weakly scattering samples. The length of the vacuum flight tube can be modified by adding or removing cylindrical elements. The standard length of these elements is 1 m. The maximum detector position inside the vacuum and from the focus is ∼4.5 m. Inside the flight tube, a pressure well below 1 mbar is routinely achieved during operation. For forward-imaging experiments, the maximal count rate of the Eiger2 detector limits the beam intensity that can be used. This limit is energy dependent, as shown in Fig. 14 ▸(*a*). Assuming that the detector is mounted at least 3.5 m downstream of the focus, the detector is able to handle the full coherent beam for all incident-beam photon energies above 12 keV. Below that energy or focus–detector distance, or in the case of partially coherent beam settings, where the flux is higher, the beam needs to be attenuated to keep the signal strength on the Eiger2 X 4M in the linear range of the detector.

For X-ray fluorescence measurements and to help with sample positioning and alignment, a 450 µm-thick one-element Si detector (RaySpec SiriusSD, Model 881-36194), with an active area of 65 mm^2^ and a 12.5 µm-thick beryllium window, is available at the endstation. It is read out by an Xspress3 digital pulse processor from Quantum Detectors and provides an energy resolution down to 133 eV (at 5.9 keV), and is able to cover the whole energy range of the beamline.

All detectors at NanoMAX can be synchronized with a fast acquisition scheme aimed at reducing dead-time between measurement points and increasing time efficiency (*cf.* Section 6).

## 6. Controls, data acquisition and visualization

Most components of the diffraction endstation along with the other beamline components of NanoMAX are interfaced via the *TANGO Controls* software (Chaize *et al.*, 1999 ▸). The data-collection and scanning schemes are orchestrated by the data-acquisition framework *Contrast* (Björling *et al.*, 2021 ▸; Björling, 2020 ▸). This provides a text-based user interface and resembles the common *SPEC* syntax that is in use at many facilities. This acquisition system is developed at the beamline, making it flexible and simple enough to be adjusted and extended as needed for user experiments. Similarly, detector pipelines are developed locally with emphasis on simplicity and performance, as described by Björling *et al.* (2020*b* ▸). Besides the software-based scanning, where the motion of the scanned axis and the acquisitions of detector frames are initiated by *Contrast*, samples can also be scanned in a continuous mode, so that the overhead of software-based scanning can be drastically reduced. In this continuous scanning mode, the sample mounted on the piezo scanner is moved at a constant speed. After a short acceleration phase, when the constant speed phase begins, a trigger is created by the controller of the piezo scanner. A PandABox (Zhang *et al.*, 2018 ▸; Christian *et al.*, 2020 ▸) receives this trigger and sends a train of trigger pulses to the detectors, which synchronizes the data acquisition. Based on the same trigger pulses, the PandABox samples the positions of the piezo scanner, so that the actual sample position can be directly associated with each detector acquisition. To map an area on the sample, a number of subsequent continuous line scans is performed. The speed of the motion and the trigger frequency are determined from a set of user-provided scan parameters, and are only limited by hardware (*i.e.* detector frame rate, minimum and maximum speed of motion). The speed of motion is not a limiting factor for the data acquisition, which is dominated by the detector frame rate. The motion within a continuous line scan is programmed as an arbitrary waveform into the piezo controller. With this flexible approach, more complex motion patterns can be realized and combined motions of several piezo axes can be obtained, as it is required for *e.g.* spiral scan paths.

**Figure 15:**
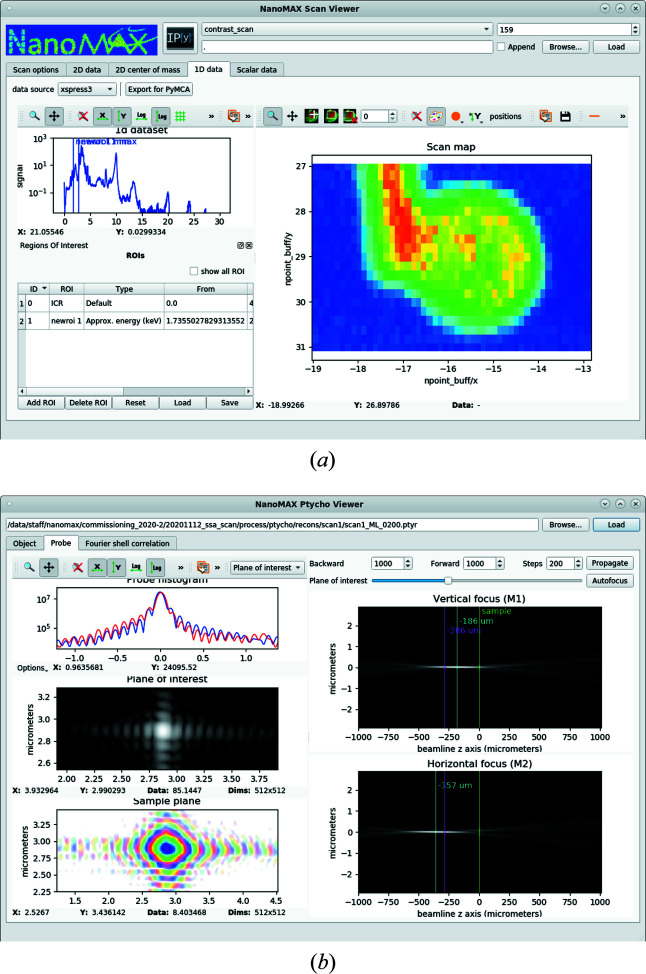
Screenshots of beamline applications for fast feedback on mapping and ptychography experiments. (*a*) A sample map corresponding to the integrated intensity in a region of the fluorescence emission spectrum. (*b*) A view on the probe as reconstructed by ptychography, along with a numerical wavefront propagation and information about the beam in focus.

A simple visualization program for fast feedback of scanning X-ray imaging experiments using pixel detectors, fluorescence or other input has been implemented, and is in continued development, since the early commissioning of the endstation (https://github.com/maxiv-science/nanomax-analysis-utils). The application is built on the *silx* suite of widgets (https://doi.org/10.5281/zenodo.591709). A screenshot with a fluorescence-mapping example from Chayanun *et al.* (2020 ▸) is shown in Fig. 15 ▸(*a*). While the program is not optimized for performance and loads data from disk only after a scan is carried out, it provides important feedback in terms of signal strengths and orientation on the sample. In it, regions of interest can be selected in pixel or emission energy and mapped out across the sample. Conversely, signals from a single point or from a region in the scan can be shown and inspected. Faster but less flexible streaming clients, which show the progress of ongoing scans, are under development.

A similar application based on *silx* has been developed to facilitate rapid feedback of ptychography reconstructions. This includes a sample view, numerical wavefront propagation and determination of the two mirrors’ focal planes relative to the sample. The latter is crucial for fast fine tuning of the KB mirror suspension and thereby adjusting their focusing. Figure 15 ▸(*b*) shows a snapshot of the application with the beam view selected.

## 7. Conclusions

The diffraction endstation of NanoMAX offers a good compromise between stability and flexibility for experiments requiring (coherent) nano-beams in the X-ray energy range between 5 and 28 keV, also in a compact sample environment. The development of sample environments is carried out in collaboration with users on the basis of their specific needs. Tailored sample environments have already been developed and successfully used at the endstation. Designed with scanning X-ray diffraction measurements in mind, this station offers a variety of complementary detection capabilities and experimental approaches, such as tomography and scanning transmission X-ray microscopy, with scattering and fluorescence signals. The excellent coherence properties of the produced nano-beam have already been exploited for 2D and 3D ptychography in forward geometry, coherent diffraction imaging of isolated particles, and 3D ptychography in Bragg. The instrument, in continued development, serves an increasingly large user community.

## Figures and Tables

**Table 1:** A summary of the range of rotations and translations available for the sample, and respective resolution

Axis	Range	Resolution
Theta rotation	−2–90°	0.1 mdeg
Phi rotation	0–360°	0.1 mdeg
*XYZ* coarse motion	±8 mm	10 nm
*XYZ* sample scanner	±50 µm	1 nm
Piezo sample rotation	0–360°	0.625 mdeg

## Footnotes

1 These numbers are based on the magnification of the optical system and the camera chip: 2× adapter – 0.57–7× zoom lens – no lens attachment with a Basler acA1300-60gc camera.
